# Phytoplasma classification and phylogeny based on *in silico* and *in vitro* RFLP analysis of *cpn60* universal target sequences

**DOI:** 10.1099/ijsem.0.001501

**Published:** 2016-12

**Authors:** Edel Pérez-López, Chrystel Y. Olivier, Mauricio Luna-Rodríguez, Tim J. Dumonceaux

**Affiliations:** ^1^​ Instituto de Biotecnología y Ecología Aplicada (INBIOTECA), Universidad Veracruzana, Avenida de Las Culturas Veracruzanas Xalapa, Veracruz, México; ^2^​ Agriculture and Agri-Food Canada, London Research and Development Centre, London, Ontario, Canada; ^3^​ Laboratorio de Alta Tecnología de Xalapa - DGI, Universidad Veracruzana, Médicos 5, Unidad del Bosque Xalapa, Veracruz, México; ^4^​ Agriculture and Agri-Food Canada, Saskatoon Research and Development Centre, Saskatoon, Saskatchewan, Canada; ^5^​ Department of Veterinary Microbiology, University of Saskatchewan, Saskatoon, Saskatchewan, Canada

**Keywords:** Phytoplasma, Diversity, differentiation, chaperonin 60, taxonomy

## Abstract

Phytoplasmas are unculturable, phytopathogenic bacteria that cause economic losses worldwide. As unculturable micro-organisms, phytoplasma taxonomy has been based on the use of the 16S rRNA-encoding gene to establish 16Sr groups and subgroups based on the restriction fragment length polymorphism (RFLP) pattern resulting from the digestion of amplicon (*in vitro*) or sequence (*in silico*) with seventeen restriction enzymes. Problems such as heterogeneity of the ribosomal operon and the inability to differentiate closely related phytoplasma strains has motivated the search for additional markers capable of providing finer differentiation of phytoplasma strains. In this study we developed and validated a scheme to classify phytoplasmas based on the use of *cpn*60 universal target (*cpn60* UT) sequences. Ninety-six *cpn*60 UT sequences from strains belonging to 19 16Sr subgroups were subjected to *in silico* RFLP using pDRAW32 software, resulting in 25 distinctive RFLP profiles. Based on these results we delineated *cpn*60 UT groups and subgroups, and established a threshold similarity coefficient for groups and subgroups classifying all the strains analysed in this study. The nucleotide identity among the reference strains, the correspondence between *in vitro* and *in silico* RFLP, and the phylogenetic relationships of phytoplasma strains based on *cpn60* UT sequences are also discussed.

Phytoplasmas, first known as mycoplasma-like organisms (Doi *et al.*, 1967), are wall-less, insect-vectored bacteria that cause disease in more than a thousand different plant hosts, affecting weedy, ornamental and crop plants worldwide (Harrison *et al.*, 2014; Pérez-López *et al.*, 2016a). With a small, A-T rich, and distinctively organized genome, phytoplasmas are a well-defined clade inside the class *Mollicutes*, derived from an *Acholeplasma*-like ancestor (Zhao *et al.*, 2014, 2015).

Phytoplasmas have not been successfully isolated in axenic cultures, so traditional taxonomic characteristics are difficult to measure and phytoplasma taxonomy remains under the classification criteria specified for uncultured micro-organisms (Murray & Stackebrandt, 1995). In 2004, the International Committee of Systematic Bacteriology Subcommittee for the Taxonomy of *Mollicutes*, the International Research Program for Comparative Mycoplasmology (IRPCM), proposed the provisional genus ‘*Candidatus* Phytoplasma’ (IRPCM, 2004). This classification is based on the similarity of 16S rRNA gene sequences supported by phylogenetic analysis, and using this strategy, 38 ‘*Candidatus* Phytoplasma’ species have been formally described to date (Davis *et al.*, 2013; Harrison *et al.*, 2014; IRPCM, 2004; Nejat *et al.*, 2013). Classification of phytoplasmas is further supported by the 16S rRNA gene through the use of restriction fragment length polymorphism (RFLP) of the 16S rRNA F2nR2 fragment with a set of seventeen endonucleases (Lee *et al.*, 1993, 1998). This approach identifies at least 30 groups of phytoplasmas, designated 16SrI-16SrXXX, with each group containing subgroups designated by letters (Harrison *et al.*, 2014; Pérez-López *et al.*, 2016a; Zhao *et al.*, 2009). The validation of a computer simulated (*in silico*) RFLP as an alternative to the actual (*in vitro*) RFLP, along with the development of the interactive online phytoplasma classification tool *i*PhyClassifier, increased the accuracy of phytoplasma classification based on 16S rRNA gene sequences (Wei *et al.*, 2007, 2008; Zhao *et al.*, 2009).

The use of other genes as part of the scheme of identification and classification of phytoplasmas has been broadly suggested, mainly because closely related strains are not well resolved using the 16S rRNA-encoding gene alone. The 16S–23S rRNA intergenic spacer, 23S rRNA region, *rp* (ribosomal protein) operon, *tuf*, *rplV* (*rpl22*)–*rpsC* (*rps3*), *secY*, *map*, *uvrB*–*degV*, *nusA, secA,* and *rpoB* genes have been used to identify and characterize phytoplasmas (Arnaud *et al.*, 2007; Botti *et al.*, 2003; Hodgetts *et al.*, 2008; Lee *et al.*, 2006; Marcone *et al.*, 2000; Shao *et al.*, 2006; Streten & Gibb, 2005; Valiunas *et al.*, 2013). All these genes have been used to achieve a finer differentiation of phytoplasmas belonging to different species and/or RFLP groups. Another gene used to improve the resolution of phytoplasmas classification is the *groEL* gene, also known as chaperonin 60 (*cpn*60) (Dumonceaux *et al.*, 2014; Mitrović *et al.*, 2011,^2015^). All the genes mentioned above have also been used to differentiate other bacterial species. Lactic acid bacteria have been differentiated and identified using RFLP analysis of *rpoB* (Claisse *et al.*, 2007), 16S rRNA/16S-23S rRNA intergenic spacer region (Ruiz *et al.*, 2000), and *tuf* (Park *et al.*, 2012). Moreover, partial *cpn60* gene sequences (500 to 550 bp), have been useful to identify novel species such as *Lactobacillus selangorensis* (Haakensen *et al.*, 2011), *Sphingobacterium detergens* (Marqués *et al.*, 2012);*Methylobacterium gnaphalii* (Tani *et al.*, 2012), and *Prevotella jejuni* (Hedberg *et al.*, 2013), among many others. The *cpn60* universal target (*cpn60* UT) (Goh *et al.*, 1996), is a fragment of approximately 550 bp that has been extensively used in the study of microbial communities (Town *et al.*, 2014), and suggested as a molecular barcode for the domain Bacteria (Links *et al.*, 2012). While not all *Mollicutes* encode Cpn60 within their genomes (Clark & Tillier, 2010), genes encoding Cpn60 have been found in all complete phytoplasma genomes reported to date and have been detected in many different phytoplasma subgroups (Andersen *et al.*, 2013; Bai *et al.*, 2006; Kube *et al.*, 2008; Oshima *et al.*, 2004; Tran-Nguyen *et al.*, 2008). However, draft genomes for phytoplasma strains from the 16SrIII group suggest that this subgroup may lack this gene (Saccardo *et al.*, 2012), which would limit the utility of *cpn60*-based classification tools for this subgroup. Nevertheless, the recent development of methods to access *cpn60* UT sequences from phytoplasmas (Dumonceaux *et al.*, 2014), has enabled the use of these sequences to develop diagnostic methods, and facilitates phytoplasma characterization based on polymorphisms detected among the different phytoplasma groups and subgroups (Dumonceaux *et al.*, 2014; Pérez-López *et al.*, 2016b). This primer cocktail has been shown to amplify the *cpn60* UT from a diverse array of phytoplasmas (sharing as little as 61 % identity at the nucleotide level) from the major groups of phytoplasmas (Chung *et al.*, 2013; Dumonceaux *et al.*, 2014), although it is acknowledged that this amplification strategy may need to be modified as new sequences accrue, particularly from genomic sequencing efforts. Moreover, nested PCR is possible using previously reported primer sets that span the *cpn60* UT of various phytoplasma groups (Kakizawa *et al.*, 2006; Mitrović *et al.*, 2011).

In this study, following the strategy previously used in the phytoplasma classification scheme based on the 16S rRNA gene, we suggest a complementary, coherent system to classify phytoplasmas based on RFLP analysis of *cpn60* UT sequences with seven endonucleases. This new classification scheme, besides being phylogenetically valid, allowed a finer differentiation of phytoplasma strains inside the same 16Sr RFLP subgroups, with the identification of *cpn60* UT groups and subgroups.

## *cpn60* UT sequences differentiate phytoplasma clade and subclades

One hundred and thirty-three *cpn60* UT sequences were retrieved from the cpnDB (Hill *et al.*, 2004) and NCBI nucleotide sequence databases. Fifty-five *cpn60* UT sequences from phytoplasma, along with three sequences belonging to Acholeplasmas, three from Mycoplasmas, one from *Clostridia*, 19 from *Bacillales*, six from *Lactobacillales*, 34 sequences from walled Gram-negative bacterial taxa (*Rhizobiales*, *Enterobacteriaceae*, *Sphingomonadales*, among others), and one sequence from Cyanobacteria used as outgroup, were aligned with clustal x version 1.63b (Thompson *et al.*, 1997) and trimmed to the 552 bp corresponding to the *cpn60* UT sequences defined for phytoplasmas (Dumonceaux *et al.*, 2014). A phylogenetic tree was reconstructed by the neighbour-joining method, using the tree-bisection-and-regrafting (TBR) algorithm available in mega6 software package (Tamura *et al.*, 2013), and was bootstrapped 1000 times. We chose neighbour-joining because this method selects pairs of taxa that decrease the overall length of the tree, and because it is computationally less intensive than other methods of calculating phylogeny (Gascuel & Steel, 2006).

**Fig. 1.**
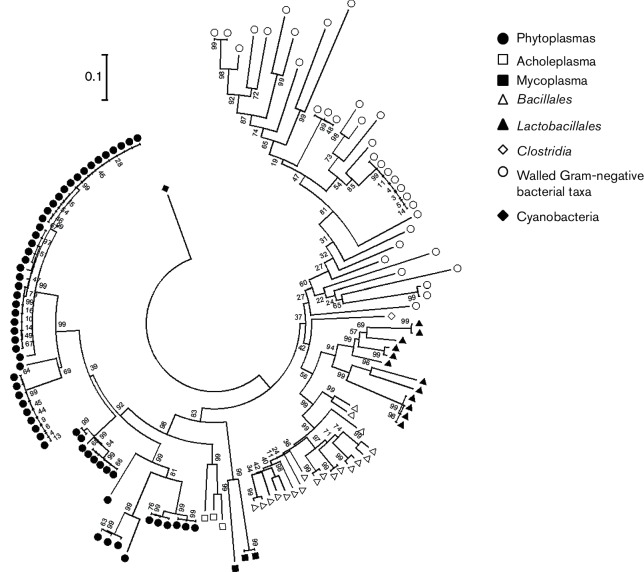
Phylogenetic tree reconstructed using the neighbour-joining method of the *cpn60* UT sequences of 163 micro-organisms within the domain Bacteria. We included 9 sequences from phytoplasma, 3 from Acholeplasmas, 3 from Mycoplasmas, 1 from *Clostridia*, 19 from *Bacillales*, 6 from *Lactobacillales*, and 34 from walled Gram-negative bacterial taxa (*Rhizobiales*, *Enterobacteriaceae*, *Sphingomonadales*). The *cpn60* UT sequence from Cyanobacteria was used as outgroup. The phylogenetic tree was bootstrapped 1000 times to achieve reliability. Bar, 1 substitution in 10 positions.

The phylogenetic tree obtained (Fig. 1) showed a clear delineation of the phytoplasma clade, with a differentiation of the three major phytoplasma subclades previously described (Chung *et al.*, 2013; Hogenhout *et al.*, 2008; Zhao *et al.*, 2010, 2014). Similar results were obtained by calculating the tree using the maximum-likelihood method (Yang, 2007) (data not shown). The tree topology corresponded with the topology previously obtained by Wei and colleagues in 2007 using 16S rRNA gene sequences (Wei *et al.*, 2007). This result confirms the ability of *cpn60* UT sequences to identify phytoplasmas through cladistics analysis, as previously suggested (Dumonceaux *et al.*, 2014; Pérez-López *et al.*, 2016b).

To identify a phytoplasma-specific ‘signature’ sequence, corresponding to that reported for the 16S rRNA-encoding gene (IRPCM, 2004), we analysed the sequences shown in Fig. 1 using sigoligo, software that can identify signature sequences (Zahariev *et al.*, 2009). This analysis revealed that the first ~60 nucleotides of the *cpn60* UT differentiated phytoplasma sequences from other *cpn60* UT sequences (data not shown). Aligning nucleotides 1–58 of all phytoplasma sequences and displaying them using Weblogo (Crooks *et al.*, 2004) suggested a possible phytoplasma-specific signature sequence (5′-GCWAYHNTWTTRGCDCAAARWATVATTCAWMRGGDTTYRAWKYDRTWRAYDYWGGDG-3′; Fig. S1, available in the online Supplementary Material) that yielded only phytoplasma sequences by fasta alignment at cpnDB (Hill *et al.*, 2004) (data not shown). Furthermore, translation of this nucleotide sequence revealed a putative, less degenerate amino acid sequence that similarly functioned as a signature sequence for phytoplasmas: [A(T/V)(V/L)LAQ(S/K/N)MI(H/R/Q)(R/K)GF(D/K)(A/F)(I/V)(D/N)(A/S/L)G; Fig. S1]. Like the nucleotide sequence, this amino acid sequence from randomly selected phytoplasmas yielded only phytoplasma sequences by blastp at cpnDB among the first 100 hits (data not shown).

## Differentiating phytoplasmas based on *cpn60* UT sequences

So far, phytoplasma *cpn60* sequences have been reported from members of the groups 16SrI, 16SrII, 16SrV, 16SrVII, 16SrIX, 16SrX, 16SrXII, 16SrXIII and 16SrXIV (Dumonceaux *et al.*, 2014; Pérez-López *et al.*, 2016b). Altogether, after trimming the *cpn60* UT sequence from the five completely sequenced phytoplasma genomes (Andersen *et al.*, 2013; Bai *et al.*, 2006; Kube *et al.*, 2008; Oshima *et al.*, 2013; Tran-Nguyen *et al.*, 2008), from the draft genome belonging to the group 16SrII-A, strain PnWB (Chung *et al.*, 2013) and 16SrIX-B strain SA213 (Quaglino *et al.*, 2015), from the *cpn60* sequences reported by Mitrović *et al.* (2011) for members of the group 16SrI, and members of the group 16SrXIV (Mitrović *et al.*, 2015), from the 3.6 kb DNA fragments obtained by Kakizawa *et al.* (2006), and the sequences previously obtained by our group, we had 96 *cpn60* UT sequences in this study.

The highest *cpn60* UT sequence diversity was observed in members of the group 16SrI, with sequences from the subgroups 16SrI- A, B, C, E, F, and P subgroups represented. We also had a *cpn60* UT sequence from more than one subgroup inside the 16Sr groups IX, X, XII and XIV. The description of the strains used and the 16Sr and suggested *cpn60*-based classifications are contained in Table S1.

**Fig. 2.**
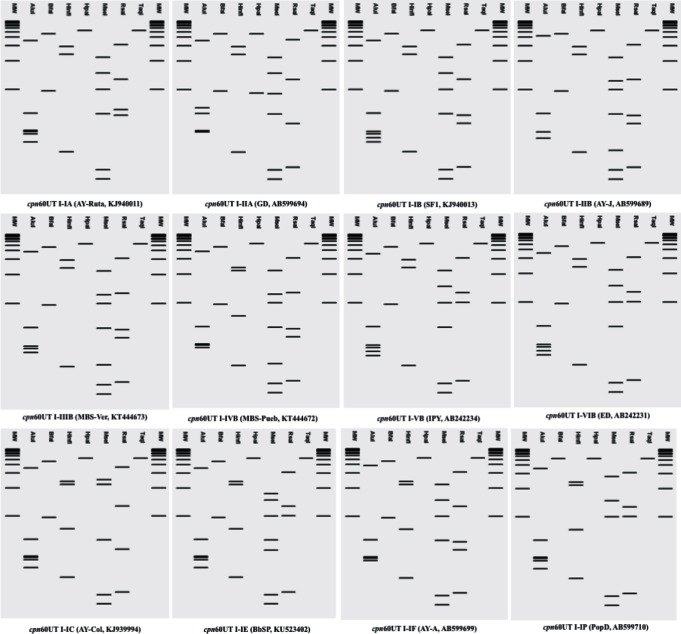
Distinctive RFLP patterns obtained with pDRAW32 from *in silico* digestion of *cpn60* UT sequence from the 12 representative *cpn60* UT subgroups within the group *cpn60* UT I. In the computer-simulated digestions, the full set of seven enzymes *Alu*I, *Bfa*I, *Hinf*I, *Hpa*I, *Mse*I, *Rsa*I and *Taq*I were used. Lanes labelled MW represent Invitrogen 1 kb plus ladder.

Since the development of the first coherent scheme to differentiate phytoplasmas, the use of RFLP has contributed to an understanding of phytoplasma diversity and has been used to differentiate strains that are phylogenetically closely related. This strategy has been used not only with the 16S rRNA gene, but also with *rp* (ribosomal protein) operon (Lee *et al.*, 1998), *sec*A (Hodgetts *et al.*, 2008), *cpn60* (Mitrović *et al.*, 2011), and recently with *rpoB* (Valiunas *et al.*, 2013). Following the strategies previously described, and taking into account the restriction sites present in the 552 bp corresponding to *cpn60* UT in phytoplasmas, we found seven endonucleases capable of differentiating phytoplasma strains. All the *cpn60* UT sequences used in this study were subjected to *in silico* RFLP with endonucleases *Alu*I, *Bfa*I, *Hinf*I, *Hpa*I, *Mse*I, *Rsa*I and *Taq*I using pDRAW32 software (AcaClone Software, http://www.acaclone.com). After comparing the RFLP patterns obtained for each strain, we detected 25 different RFLP patterns from 19 16Sr subgroups, which points to the increased diversity observed using *cpn60* UT as an additional marker to differentiate phytoplasmas. The highest diversity was detected inside the 16SrI group. We detected two *cpn60* RFLP profiles among the strain members of the 16SrI-A subgroup and six distinctive RFLP profiles within the members of the 16SrI-B subgroup, while for the rest of the subgroups we detected only one *cpn60* RFLP pattern for each corresponding 16Sr subgroup. The virtual 4 % agarose gel electrophoresis patterns observed for each of the 25 reference strains detected in this study are presented in Figs 2 and3.

**Fig. 3.**
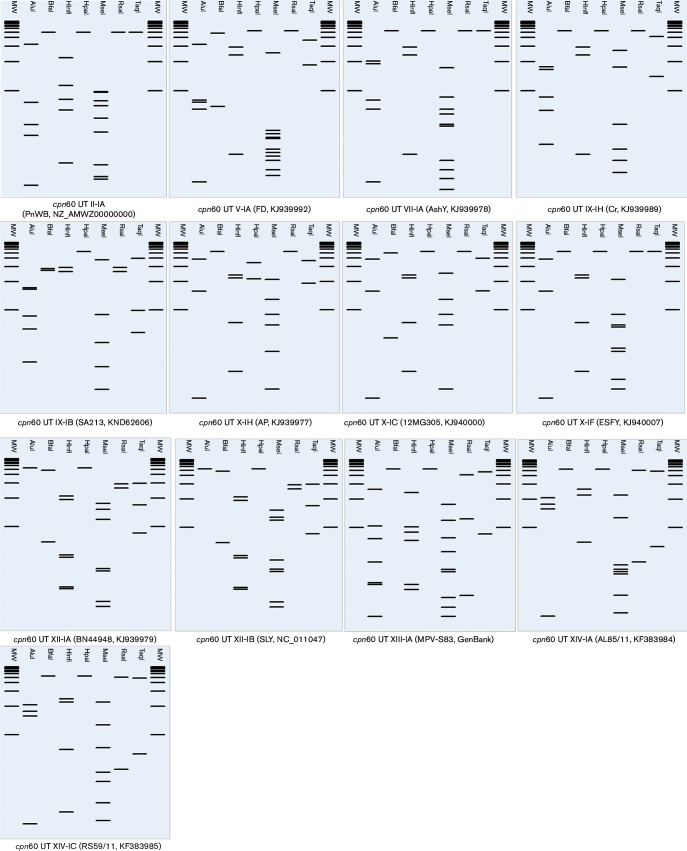
Distinctive RFLP pattern obtained with pDRAW32 from *in silico* digestion of *cpn60* UT sequence from the 12 representative *cpn60* UT subgroups within the groups *cpn60* UT II, *cpn60* UT V, *cpn60* UT VII, *cpn60* UT IX, *cpn60* UT X, *cpn60* UT XII, *cpn60* UT XIII and *cpn60* UT XIV. In the computer-simulated digestions, the set of seven enzymes *Alu*I, *Bfa*I, *Hinf*I, *Hpa*I, *Mse*I, *Rsa*I and *Taq*I were used. Lanes labelled MW represent Invitrogen 1 kb plus ladder.

Based on the RFLP patterns observed, we separated the strains into *cpn60* UT-based subgroups. To maintain consistency with the established nomenclature based on the 16S rRNA-encoding gene, we named the strains from group 16SrI as *cpn60* UT I, 16SrII as *cpn60* UT II, and so on. To name subgroups, for example the 16SrI-B, which had until now six different RFLP patterns among strains, we named the *cpn60* UT subgroups as *cpn60* UT I-IB, *cpn60* UT I-IIB, *cpn60* UT I-IIIB, (…), *cpn60* UT I-VIB. All 96 strains analysed in this study were reclassified based on their *cpn60* UT RFLP patterns (Table S1).

To establish the threshold similarity coefficient to delineate new *cpn60* UT groups and subgroups, we calculated the similarity coefficients (F) among the 25 reference strains with unique RFLP patterns. We used the formula F=2N*xy* / (N*x*+N*y*) (Nei & Li, 1979), where N*x* and N*y* are the number of bands resulting from the digestion of *cpn60* UT with the seven endonucleases for strain x and strain y, respectively, and N*xy* is the number of bands common to both strains. The number of bands generated by digesting the reference *cpn60* UT sequences with each of the seven endonucleases used in this study is shown in Table 1.

**Table 1.** Number of bands produced by RFLP analysis of *cpn60* UT sequences from reference phytoplasma strains of *cpn60* UT groups.

		No. of bands generated						
*cpn60* UT group	Strain	*Alu*I	*Bfa*I	*Hinf*I	*Hpa*I	*Mse*I	*Rsa*I	*Taq*I
***cpn60* UT I**								
*cpn60* UT I-IA	AY-Ruta	6	2	3	1	6	4	1
*cpn60* UT I-IIA	GD	5	2	3	2	7	4	1
*cpn60* UT I-IB	SF1	7	2	3	1	6	5	1
*cpn60* UT I-IIB	AY-J	5	2	3	1	7	5	1
*cpn60* UT I-IIIB	MBS-Ver	6	2	3	1	7	5	1
*cpn60* UT I-IVB	MBS-Pueb	5	2	4	1	7	5	1
*cpn60* UT I-VB	IPY	7	2	3	1	6	4	1
*cpn60* UT I-VIB	ED	6	2	3	1	6	4	1
*cpn60* UT I-IC	AY-Col	6	*2*	4	1	5	4	1
*cpn60* UT I-IE	BbSP	6	2	4	1	7	4	1
*cpn60* UT I-IF	AY-A	5	2	4	1	6	5	1
*cpn60* UT I-IP	PopD	6	1	4	1	5	4	1
***cpn60* UT II**								
*cpn60* UT II-IA	PnWB	6	1	5	1	9	1	1
***cpn60* UT V**								
*cpn60* UT V-IA	FD	5	2	3	1	14	1	3
***cpn60* UT VII**								
*cpn60* UT VII-IA	AshY	5	1	3	1	12	1	1
***cpn60* UT IX**								
*cpn60* UT IX-IH	Cr	6	1	3	1	6	1	2
*cpn60* UT IX-IB	SA213	6	2	3	1	5	2	3
***cpn60* UT X**								
*cpn60* UT X-IA	AP	3	1	4	2	6	1	2
*cpn60* UT X-IC	12MG305	3	2	4	1	5	1	2
*cpn60* UT X-IF	ESFY	3	1	4	1	8	1	1
***cpn60* UT XII**								
*cpn60* UT XII-IA	BN44948	1	2	6	1	7	2	3
*cpn60* UT XII-IB	AT	1	2	6	1	8	2	3
***cpn60* UT XIII**								
*cpn60* UT XIII-IA	MPV-S83	7	1	6	1	9	3	2
***cpn60* UT XIV**								
*cpn60* UT XIV-IA	AL85/11	4	1	3	1	10	2	2
*cpn60* UT XIV-IC	RS59/11	4	1	4	1	9	2	2

The similarity coefficients among the 25 reference strains are shown in Table 2. We found that the F value between strains from the same *cpn60* UT group varied from 0.97 to 0.62, while F values lower than 0.62 belonged to strains classified in a different *cpn60* UT group (Table 2). Based on these results we confirmed the presence of two *cpn60* UT subgroups inside the *cpn60* UT I-A group (*cpn60* UT I-IA and *cpn60* UT I-IIA), and six subgroups inside the *cpn60* UT I-B group (*cpn60* UT I-IB to *cpn60* UT I-VIB). We suggest 0.97 as the threshold similarity coefficient to delineate new subgroups based on the use of the seven endonucleases previously mentioned, while 0.60 can be considered as the threshold similarity coefficient to delineate new groups. The threshold to delineate new *cpn60* UT subgroups (0.97), corresponds with the threshold to delineate new 16S rRNA gene subgroups (Wei *et al.*, 2007).

Subgroup *cpn60* UT I-IA is represented by *Brassica spp.* phytoplasma strain AY-Ruta (GenBank accession no. KJ940011), and *cpn60* UT I-IIA is represented by Grey dogwood stunt phytoplasma strain GD (GenBank accession no. AB599694). The subgroup *cpn60* UT I-IB is represented by *Linum usitatissimum* phytoplasma strain SF1 (GenBank accession no. KJ940013); *cpn60* UT I-IIB is represented by Aster yellow phytoplasma strain AY-J (GenBank accession no. AB599689); *cpn60* UT I-IIIB and *cpn60* UT I-IVB are represented by Maize bushy stunt phytoplasma, strains MBS-Ver (GenBank accession no. KT444673) and MBS-Pueb (GenBank accession no. KT444672), respectively. Subgroup *cpn60* UT I-VB is represented by Iceland poppy yellows phytoplasma strain IPY (GenBank accession no. AB242234), and the subgroup *cpn60* UT I-VIB is represented by Eggplant dwarf phytoplasma strain ED (GenBank accession no. AB242231). Subgroup *cpn60* UT I-IC is represented by Aster Yellow phytoplasma strain AY-Col (GenBank accession no. KJ939994); *cpn60* UT I-IE is represented by Blueberry stunt phytoplasma strain BbSP (GenBank accession no. KU523402); *cpn60* UT I-IF is represented by Apricot chlorotic leafroll phytoplasma strain AY-A (GenBank accession no. AB599699); and *cpn60* UT I-IP represented by Populus decline phytoplasma strain PopD (GenBank accession no. AB599710).

Inside the groups *cpn60* UT II, V, VII, and XIII, we only had strains from one subgroup, so we were not able to detect more than one RFLP pattern. The subgroup *cpn60* UT II-IA is represented by Peanut witches’-broom phytoplasma strain PnWB (GenBank accession no. NZ_AMWZ00000000); subgroup *cpn60* UT V-IA is represented by the *Flavescence doree* phytoplasma strain FD (GenBank accession no. KJ939992); the subgroup *cpn60* UT VII-IA, on the other hand, is represented by Ash Yellow phytoplasma strain AshY (GenBank accession no. KJ939978). Subgroup *cpn60* UT IX-IH and *cpn60* UT IX-IB are represented by *Catharanthus roseus* phoenicium phytoplasma strain Cr (GenBank accession no. KJ939989) and Almond witches’-broom strain SA213 (GenBank accession no. KND62606), respectively. Inside the group *cpn60* UT X, we were able to differentiate members of the subgroups *cpn60* UT X-IA, represented by Apple proliferation phytoplasma (GenBank accession no. KJ939977), members of the subgroup *cpn60* UT X-IC represented by Pear decline phytoplasma strain 12MG305 (GenBank accession no. KJ940000), and members of the subgroup *cpn60* UT X-IF represented by the European stone fruit phytoplasma strain ESFY (GenBank accession no. KJ940007). Inside the group *cpn60* UT XII we identified two subgroups, subgroup *cpn60* UT XII-IA, represented by Bois noir phytoplasma strain BN44948 (GenBank accession no. KJ939979), and subgroup *cpn60* UT XII-IB, represented by Strawberry lethal yellow strain AT (GenBank accession no. NC_011047). Subgroup *cpn60* UT XIII-IA was represented by Mexican periwinkle virescence strain MPV-S83 (GenBank accession no. KT444668). Finally, we identified two subgroups inside the group *cpn60* UT XIV, subgroup *cpn60* UT XIV-IA, represented by Bermuda white leaf phytoplasma strain AL85/11 (GenBank accession no. KF383984), and subgroup *cpn60* UT XIV-IC represented by Bermuda white leaf phytoplasma strain RS59/11 (GenBank accession no. KF383985).

**Fig. 4.**
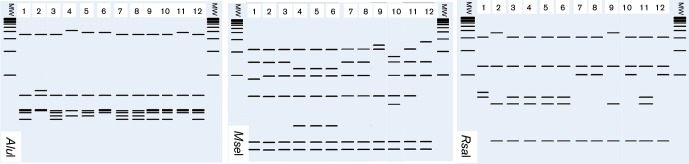
Key restriction enzymes to differentiate strains belonging to the subgroups within the group *cpn60* UT I. Lanes 1 and 2 represent subgroups *cpn60* UT I-IA and *cpn60* UT I-IIA, respectively. Lanes 3 to 8 represent strains *cpn60* UT I-IB to *cpn60* UT I-VIB, respectively. Lanes 9, 10, 11, and 12 represent strains *cpn60* UT I-IC, *cpn60* UT I-IE, *cpn60* UT I-IF, and *cpn60* UT I-IP, respectively. Lanes labelled MW represent Invitrogen 1 kb plus ladder.

**Fig. 5.**
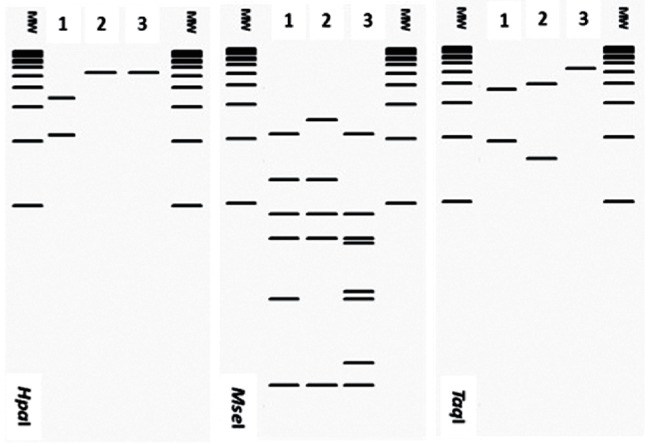
Key restriction enzymes to differentiate strains belonging to the subgroups within the group *cpn60* UT X. Lanes 1, 2, and 3 represent subgroups *cpn60* UT X-IA, *cpn60* UT X-IC, and *cpn60* UT X-IF, respectively. Lanes labelled MW represent Invitrogen 1 kb plus ladder.

**Fig. 6.**
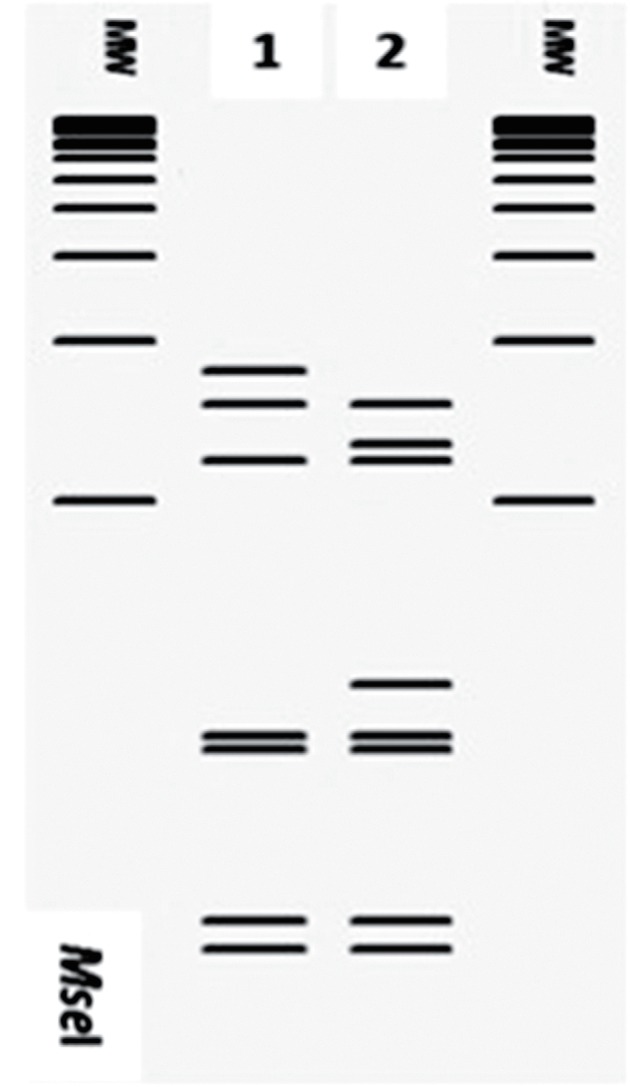
Key restriction enzymes to differentiate strains belonging to the subgroups within the group *cpn60* UT XII. Lanes 1 and 2 represent subgroups *cpn60* UT XII-IA and *cpn60* UT XII-IB, respectively. Lanes labelled MW represent Invitrogen 1 kb plus ladder.

**Fig. 7.**
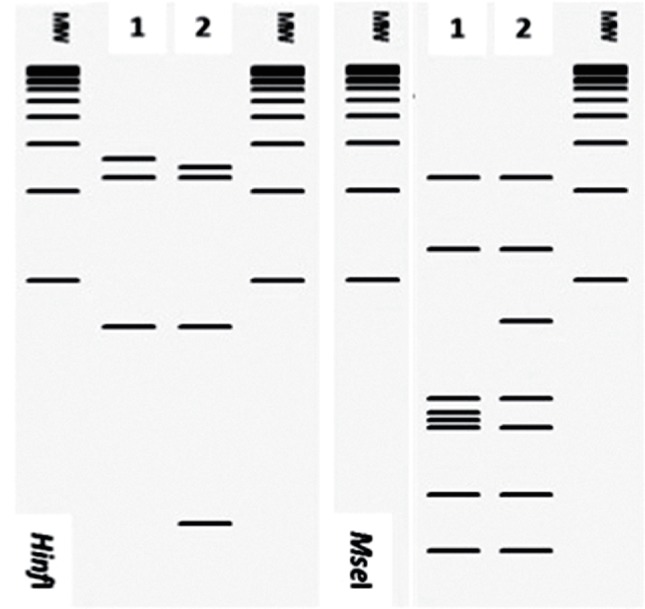
Key restriction enzymes to differentiate strains belonging to the subgroups within the group *cpn60* UT XIV. Lanes 1 and 2 represent subgroups *cpn60* UT XIV-IA and *cpn60* UT XIV-IC, respectively. Lanes labelled MW represent Invitrogen 1 kb plus ladder.

Analysing the RFLP patterns for each group, we identified enzymes capable of differentiating *cpn60* UT-subgroups. Subgroups from the group *cpn60* UT I can be differentiated through the use of *Alu*I, *Mse*I and *Rsa*I (Fig. 4). Subgroups from group *cpn60* UT X can be differentiated using endonucleases *Hpa*I, *Mse*I and *Taq*I (Fig. 5). Subgroups included in group *cpn60* UT XII can be differentiated only by the pattern generated by *Mse*I (Fig. 6), while subgroups within *cpn60* UT XIV can be differentiated by *Hinf*I and *Mse*I (Fig. 7). The *in vitro* RFLP profile from strains within the group *cpn60* UT IX differed with six of the seven endonucleases (not shown). Moreover, we observed correspondence between the *in silico* and *in vitro* RFLP for 12 phytoplasma strains representing the three major phylogenetic subclades into which phytoplasmas are grouped [(Dumonceaux *et al.*, 2014); not shown].

After aligning the 25 *cpn60* UT reference strains we detected 92–99 % nucleotide sequence identity among *cpn60* UT subgroups within the same group, while the sequence identities between groups was 61–84 %. The variability shown by *cpn60* UT sequences was higher compared to the 16Sr RNA gene and other genes previously used as phytoplasma markers. *cpn60* UT sequences could differentiate closely related phytoplasma strains more precisely. We observed the same trend between similarity coefficient (Table 2), and nucleotide similarity (Table 3).

**Table 2.** Similarity coefficients obtained from RFLP analysis of *cpn60* UT sequences from reference phytoplasma strains

	*cpn60* UT classification	Strains	1	2	3	4	5	6	7	8	9	10	11	12	13	14	15	16	17	18	19	20	21	22	23	24
1	*cpn60* UT I-IA	AY-Ruta	1.00																							
2	*cpn60* UT I-IIA	GD	0.83	1.00																						
3	*cpn60* UT I-IB	SF1	0.95	0.84	1.00																					
4	*cpn60* UT I-IIB	AY-J	0.86	0.81	0.92	1.00																				
5	*cpn60* UT I-IIIB	MBS-Ver	0.87	0.79	0.95	0.97	1.00																			
6	*cpn60* UT I-IVB	MBS-Pueb	0.89	0.79	0.90	0.92	0.95	1.00																		
7	*cpn60* UT I-VB	IPY	0.89	0.81	0.97	0.89	0.92	0.89	1.00																	
8	*cpn60* UT I-VIB	ED	0.94	0.83	0.95	0.86	0.89	0.92	0.97	1.00																
9	*cpn60* UT I-IC	AY-Col	0.89	0.83	0.89	0.81	0.84	0.86	0.86	0.89	1.00															
10	*cpn60* UT I-IE	BbSP	0.89	0.79	0.90	0.82	0.85	0.87	0.92	0.95	0.85	1.00														
11	*cpn60* UT I-IF	AY-A	0.92	0.81	0.92	0.89	0.92	0.95	0.89	0.92	0.92	0.92	1.00													
12	*cpn60* UT I-IP	PopD	0.83	0.72	0.84	0.76	0.79	0.81	0.86	0.89	0.83	0.89	0.81	1.00												
13	*cpn60* UT II-IA	PnWB	0.15	0.10	0.15	0.15	0.15	0.15	0.15	0.15	0.15	0.15	0.15	0.21	1.00											
14	*cpn60* UT IX-IA	Cr	0.41	0.29	0.39	0.29	0.33	0.34	0.40	0.41	0.41	0.39	0.34	0.47	0.22	1.00										
15	*cpn60* UT V-IA	FD	0.29	0.19	0.27	0.23	0.27	0.28	0.28	0.29	0.29	0.27	0.28	0.24	0.13	0.35	1.00									
16	*cpn60* UT VII-IA	AshY	0.26	0.15	0.24	0.20	0.24	0.30	0.25	0.26	0.26	0.29	0.25	0.31	0.24	0.38	0.58	1.00								
17	*cpn60* UT X-IA	AP	0.31	0.31	0.29	0.30	0.29	0.30	0.30	0.31	0.38	0.35	0.36	0.38	0.17	0.40	0.42	0.34	1.00							
18	*cpn60* UT X-IC	12MG305	0.39	0.32	0.36	0.38	0.36	0.38	0.38	0.39	0.45	0.42	0.44	0.39	0.18	0.41	0.38	0.35	0.74	1.00						
19	*cpn60* UT X-IF	ESFY	0.41	0.35	0.39	0.40	0.39	0.40	0.40	0.41	0.47	0.44	0.46	0.47	0.27	0.44	0.45	0.43	0.80	0.76	1.00					
20	*cpn60* UT XII-IA	BN44948	0.29	0.23	0.27	0.28	0.27	0.28	0.28	0.29	0.29	0.27	0.28	0.29	0.11	0.36	0.29	0.21	0.32	0.33	0.36	1.00				
21	*cpn60* UT XII-IB	SYL	0.28	0.22	0.26	0.27	0.26	0.27	0.27	0.28	0.28	0.26	0.27	0.28	0.10	0.35	0.29	0.26	0.31	0.32	0.35	0.97	1.00			
22	*cpn60* UT XIII-IA	MPV-S83	0.38	0.38	0.40	0.42	0.41	0.42	0.42	0.43	0.48	0.45	0.47	0.48	0.13	0.35	0.33	0.27	0.47	0.38	0.50	0.49	0.48	1.00		
23	*cpn60* UT XIV-IA	AL85/11	0.17	0.11	0.16	0.16	0.16	0.16	0.16	0.17	0.17	0.16	0.16	0.22	0.21	0.35	0.24	0.26	0.38	0.32	0.41	0.29	0.28	0.33	1.00	
24	*cpn60* UT XIV-IC	RS59/11	0.17	0.11	0.16	0.16	0.16	0.16	0.16	0.17	0.16	0.16	0.16	0.22	0.21	0.35	0.19	0.26	0.31	0.32	0.35	0.23	0.22	0.29	0.94	1.00

**Table 3.** Nucleotide similarity obtained from the alignment of *cpn60* UT sequences from reference phytoplasma strains

	*cpn60* UT classification	Strains	1	2	3	4	5	6	7	8	9	10	11	12	13	14	15	16	17	18	19	20	21	22	23	24
1	*cpn60* UT I-IA	AY-Ruta	1																							
2	*cpn60* UT I-IIA	GD	0.98	1																						
3	*cpn60* UT I-IB	SF1	0.97	0.97	1																					
4	*cpn60* UT I-IIB	AY-J	0.97	0.97	0.99	1																				
5	*cpn60* UT I-IIIB	MBS-Ver	0.97	0.97	0.99	0.99	1																			
6	*cpn60* UT I-IVB	MBS-Pueb	0.97	0.97	0.99	0.99	0.99	1																		
7	*cpn60* UT I-VB	IPY	0.97	0.97	0.99	0.99	0.99	0.99	1																	
8	*cpn60* UT I-VIB	ED	0.97	0.97	0.99	0.99	0.99	0.99	0.99	1																
9	*cpn60* UT I-IC	AY-Col	0.98	0.98	0.97	0.97	0.97	0.97	0.97	0.97	1															
10	*cpn60* UT I-IE	BbSP	0.97	0.97	0.97	0.96	0.97	0.97	0.97	0.97	0.98	1														
11	*cpn60* UT I-IF	AY-A	0.97	0.97	0.97	0.96	0.97	0.97	0.97	0.97	0.98	0.98	1													
12	*cpn60* UT I-IP	PopD	0.94	0.94	0.94	0.93	0.94	0.94	0.94	0.94	0.94	0.94	0.94	1												
13	*cpn60* UT II-IA	PnWB	0.65	0.65	0.65	0.65	0.65	0.65	0.65	0.65	0.65	0.65	0.66	0.65	1											
14	*cpn60* UT IX-IA	Cr	0.7	0.7	0.7	0.7	0.7	0.7	0.7	0.7	0.7	0.71	0.7	0.69	0.66	1										
15	*cpn60* UT V-IA	FD	0.66	0.66	0.65	0.65	0.65	0.65	0.65	0.65	0.66	0.66	0.66	0.66	0.64	0.69	1									
16	*cpn60* UT VII-IA	AshY	0.62	0.63	0.62	0.62	0.62	0.62	0.62	0.62	0.63	0.63	0.63	0.63	0.63	0.7	0.83	1								
17	*cpn60* UT X-IA	AP	0.76	0.76	0.76	0.76	0.76	0.76	0.76	0.76	0.76	0.76	0.76	0.75	0.64	0.74	0.71	0.69	1							
18	*cpn60* UT X-IC	12MG305	0.75	0.76	0.76	0.76	0.76	0.76	0.76	0.76	0.76	0.75	0.76	0.76	0.64	0.73	0.7	0.69	0.94	1						
19	*cpn60* UT X-IF	ESFY	0.77	0.78	0.78	0.78	0.78	0.78	0.78	0.78	0.78	0.78	0.78	0.77	0.65	0.74	0.71	0.69	0.95	0.95	1					
20	*cpn60* UT XII-IA	BN44948	0.8	0.79	0.8	0.8	0.8	0.8	0.8	0.8	0.8	0.79	0.79	0.81	0.63	0.69	0.61	0.61	0.74	0.74	0.75	1				
21	*cpn60* UT XII-IB	SYL	0.8	0.79	0.8	0.8	0.8	0.8	0.8	0.8	0.8	0.79	0.79	0.81	0.63	0.68	0.61	0.61	0.74	0.74	0.75	0.99	1			
22	*cpn60* UT XIII-IA	MPV-S83	0.82	0.82	0.82	0.82	0.82	0.82	0.82	0.82	0.83	0.82	0.82	0.84	0.64	0.71	0.65	0.64	0.76	0.78	0.78	0.81	0.81	1		
23	*cpn60* UT XIV-IA	AL85/11	0.69	0.69	0.7	0.7	0.7	0.7	0.7	0.7	0.69	0.7	0.7	0.7	0.67	0.75	0.72	0.71	0.76	0.76	0.76	0.67	0.67	0.69	1	
24	*cpn60* UT XIV-IC	RS59/11	0.68	0.69	0.7	0.7	0.7	0.7	0.7	0.7	0.69	0.7	0.69	0.69	0.67	0.75	0.71	0.7	0.75	0.75	0.76	0.67	0.67	0.68	0.96	1

**Fig. 8.**
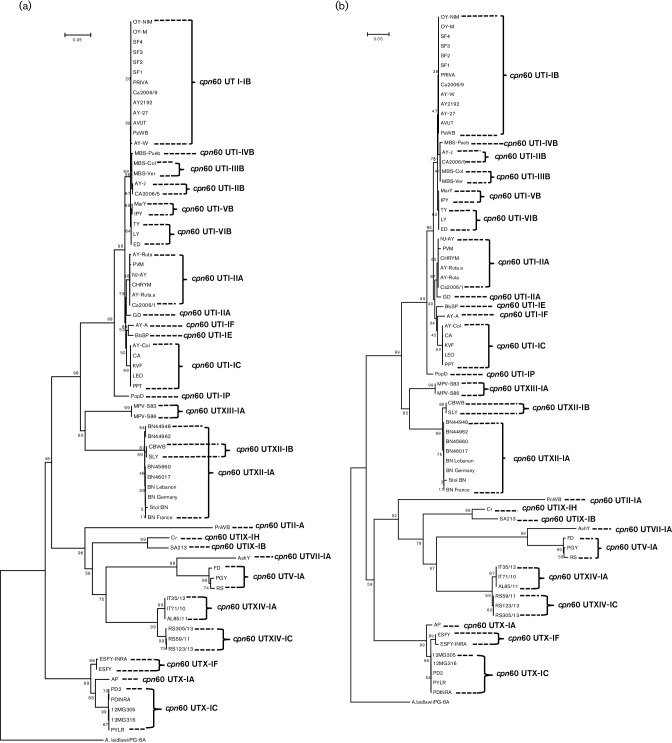
Phylogenetic tree reconstructed through the neighbuor-joining method of the *cpn60* UT nucleotide (a) and amino acid (b) sequences of phytoplasma strains from the *cpn60* groups and subgroups described in this study. Strain descriptions and GenBank accession numbers are shown in Table S1. *Acholeplasma laidlawii* was used as outgroup. Trees were bootstrapped 1000 times to achieve reliability. Bar, 5 substitutions in 100 positions.

Phylogenetic analysis of *cpn60* UT sequences of all the groups and subgroups identified in this study was performed using the neighbour-joining method, using the tree-bisection-and-regrafting (TBR) algorithm available in the mega6 software package (Tamura *et al.*, 2013), with bootstrapping 1000 times for nucleotide (Fig. 8a) and amino acid (Fig. 8b) sequences. Both phylogenetic trees showed distinction between the *cpn60* UT groups and subgroups, supporting the results obtained through the RFLP analysis, the calculation of F value and the nucleotide identity among the reference strains. Phylogenetic analysis of *cpn60* UT sequences showed a better resolution of the subgroup B, identified inside the group *cpn60* UT I (Fig. 8a), while the phylogenetic tree using the amino acid sequences allowed a better resolution of the subgroups identified within the group *cpn60* UT XII (Fig. 8b).

The present study confirms previously published work (Dumonceaux *et al.*, 2014; Mitrović *et al.*, 2011, 2015) showing the capability of *cpn60* UT sequences to act as an additional marker to differentiate phytoplasmas. Strains that are closely related based on 16S rRNA gene sequence classification were differentiated as members of new subgroups, contributing to a better identification of the strains. Previous studies mentioned a high nucleotide similarity between the *cpn60-*encoding genes amplified from members of the 16SrI-B subgroup(Kakizawa *et al.*, 2006), but with the increased number of the strains characterized in this study, we showed that the nucleotide variability is higher among strains from the same 16Sr subgroup than was thought.

Protein-encoding genes are known to provide a better strain resolution compared to rRNA-encoding genes (Zeigler, 2003). Unlike the 16S rRNA gene, *cpn60* is present in a single copy in the phytoplasma genome, which obviates the taxonomic complications related with the occasional presence of heterogeneous ribosomal operons (Wei *et al.*, 2007; Zhao *et al.*, 2009). The identification of distinct phytoplasma strains is very important to vector studies, epidemiological research and development of management strategies. The classification scheme we describe herein provides a supplementary tool to the existing classification scheme based on the 16S rRNA-based F2nR2 locus. If certain subgroups of phytoplasma are confirmed to lack a gene encoding Cpn60, then this classification scheme will not apply to these groups. However, it has been noted that Mollicutes lacking *cpn60* do not tend to invade cells (Clark & Tillier, 2010), so phytoplasmas that do not encode this gene would constitute exceptions among the Mollicutes. Nevertheless, including *cpn60* UT among the additional markers used to characterize phytoplasma strains will improve the understanding of phytoplasmas. This study, supported by the cpnDB (Hill *et al.*, 2004), could be the first step in the development of interactive online tools capable of classifying phytoplasmas based on an unknown *cpn60* UT sequence amplified from phytoplasmas.

## Supplementary Data

454 Supplementary File 1
