# Supplementary material for: Phytoplasma classification and phylogeny based on in silico and in vitro RFLP analysis of cpn60 universal target sequences
Source: Int J Syst Evol Microbiol. 2016 Dec;66(12):5600–13. doi: 10.1099/ijsem.0.001501 (PMC5244502; doi:10.1099/ijsem.0.001501)
Supplement: Supplementary File 1 [file ijsem-66-1501-s001.pdf]

Phytoplasma classification and phylogeny based on *in silico* and *in vitro* RFLP analysis of *cpn60* universal target sequences

Edel Pérez-López [1], Chrystel Y. Olivier [2], Mauricio Luna-Rodríguez[3] and Tim J. Dumonceaux [4,5]

1- Instituto de Biotecnología y Ecología Aplicada (INBIOTECA), Universidad Veracruzana, Avenida de Las Culturas Veracruzanas, Xalapa, Veracruz, México.

2- Agriculture and Agri-Food Canada, London Research and Development Centre, London, Ontario, Canada.

3- Laboratorio de Alta Tecnología de Xalapa - DGI, Universidad Veracruzana, Médicos 5, Unidad del Bosque, Xalapa, Veracruz, México.

4- Agriculture and Agri-Food Canada, Saskatoon Research and Development Centre, Saskatoon, Saskatchewan, Canada.

5- Department of Veterinary Microbiology, University of Saskatchewan, Saskatoon, Saskatchewan, Canada.

Corresponding author: Tim J. Dumonceaux; **E-mail:** tim.dumonceaux@agr.gc.ca; **Tel:** 1-306-385-9450; **Fax:** 1-306-385-9482

International Journal of Systematic and Evolutionary Microbiology

Supplemental information

A

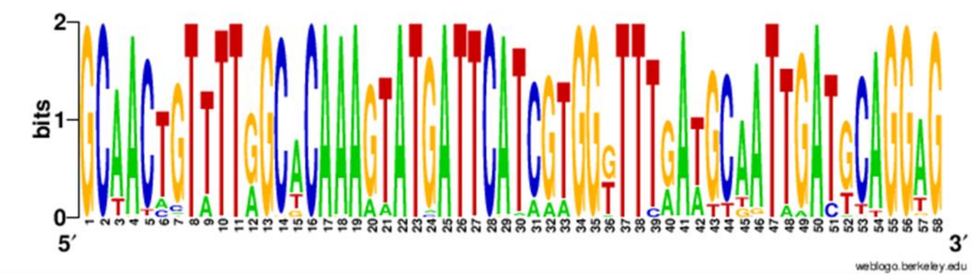

B

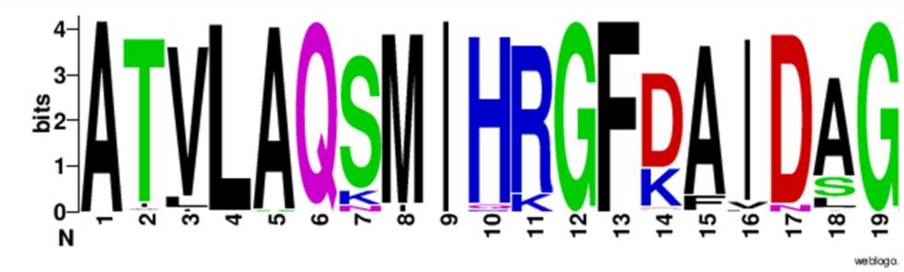

Fig. S1. Determination of a phytoplasma "signature" sequence based on the *cpn60* UT. Phytoplasma sequences were compared with related sequences (Fig. 1) using sigoligo (Zahariev *et al.*, 2009), revealing that the first ~60 nucleotides corresponded to a "signature" for phytoplasma sequences. The first 58 nucleotides were aligned and displayed using Weblogo (Crooks *et al.*, 2004) at the nucleotide (A) and amino acid (B) levels to determine a "signature" *cpn60* subsequence for phytoplasmas.

References

Crooks, G. E., Hon, G., Chandonia, J.-M. & Brenner, S. E. (2004). WebLogo: A Sequence Logo Generator. *Genome Res* **14**, 1188-1190.

Zahariev, M., Dahl, V., Chen, W. & Levesque, C. A. (2009). Efficient algorithms for the discovery of DNA oligonucleotide barcodes from sequence databases. *Mol Ecol Resour* **9 Suppl s1**, 58-64.

Table S1. Classification of phytoplasma strains based on RFLP analysis of cpn60 UT sequences.

16Sr group-subgroup	Strain	Original source	*cpn60* UT group-subgroup	GenBank accession no./ cpnDB accession no.	Reference
**16SrI**					
16SrI-A	*Brassica spp.* Phytoplasma (AY-Ruta)[RS]	Canada	*cpn60* UT I-IA	KJ940011/ b27094	Dumonceaux et al. (2014)
16SrI-A	*Allium cepa* phytoplasma (O2)	Canada	*cpn60* UT I-IA	KJ939997/ b27095	Dumonceaux et al. (2014)
16SrI-A	*Allium cepa* phytoplasma (O2L)	Canada	*cpn60* UT I-IA	KJ939998/ b27096	Dumonceaux et al. (2014)
16SrI-A	*Allium cepa* phytoplasma (O2LMB)	Canada	*cpn60* UT I-IA	KJ939999/ b27097	Dumonceaux et al. (2014)
16SrI-A	Ligustrum sinense phytoplasma (PRIVET)	Canada	*cpn60* UT I-IA	KJ940009/ b27098	Dumonceaux et al. (2014)
16SrI-A	*Brassica spp.* Phytoplasma (AY-Ruta.a)	Canada	*cpn60* UT I-IA	KJ939986/ b27099	Dumonceaux et al. (2014)
16SrI-A	Carrot yellows (ca2006/1)	Serbia	*cpn60* UT I-IA	AB599708/ b27100	Mitrović et al. (2011)
16SrI-A	Chrysanthemum yellows (CHRYM)	Germany	*cpn60* UT I-IA	AB599692/ b27101	Mitrović et al. (2011)
16SrI-A	New Jersey aster yellow (NJ-AY)	USA	*cpn60* UT I-IA	AB599703/ b27102	Mitrović et al. (2011)
16SrI-A	Plantago virescence (PVM)	Germany	*cpn60* UT I-IA	AB599706/ b27103	Mitrović et al. (2011)
16SrI-A	Aster yellows witches'-broom phytoplasma (AYWB)	USA	*cpn60* UT I-IA	NC_007716/b8392	Bai et al. (2006)
16SrI-A	Grey dogwood stunt (GD)[RS]	USA	*cpn60* UT I-IIA	AB599694/ b27104	Mitrović et al. (2011)
16SrI-B	*Linum usitatissimum* phytoplasma (SF1)[RS]	Canada	*cpn60* UT I-IB	KJ940013/ b27105	Dumonceaux et al. (2014)
16SrI-B	*Linum usitatissimum* phytoplasma (SF2)	Canada	*cpn60* UT I-IB	KJ940014/ b27106	Dumonceaux et al. (2014)
16SrI-B	*Linum usitatissimum* phytoplasma (SF3)	Canada	*cpn60* UT I-IB	KJ940015/ b27107	Dumonceaux et al. (2014)
16SrI-B	*Linum usitatissimum* phytoplasma (SF4)	Canada	*cpn60* UT I-IB	KJ940016/ b27108	Dumonceaux et al. (2014)
16SrI-B	*Thlapsi arvense* phytoplasma (SWR1)	Canada	*cpn60* UT I-IB	KJ940017/ b27109	Dumonceaux et al. (2014)
16SrI-B	*Thlapsi arvense* phytoplasma (SWR2)	Canada	*cpn60* UT I-IB	KJ940018/ b27110	Dumonceaux et al. (2014)
16SrI-B	Periwinkle aster yellow (P-AY)	Canada	*cpn60* UT I-IB	KJ940004/ b27111	Dumonceaux et al. (2014)
16SrI-B	Apricot atypical aster yellow (AVUT)	Germany	*cpn60* UT I-IB	AB599686/ b27112	Mitrović et al. (2011)
16SrI-B	Aster yellow (AY-27)	Canada	*cpn60* UT I-IB	AB599688/ b27113	Mitrović et al. (2011)
16SrI-B	Aster yellow (A2192)	Germany	*cpn60* UT I-IB	AB599687/ b27114	Mitrović et al. (2011)
16SrI-B	Primrose virescence (PRIVA)	Germany	*cpn60* UT I-IB	AB599705/ b27115	Mitrović et al. (2011)
16SrI-B	Diplotaxis virescence (DIV)	Spain	*cpn60* UT I-IB	AB599693/ b27116	Mitrović et al. (2011)
16SrI-B	Primula green yellows (PrG)	UK	*cpn60* UT I-IB	AB599696/ b27117	Mitrović et al. (2011)
16SrI-B	Oilseed rape virescence (RV)	France	*cpn60* UT I-IB	AB599698/ b27118	Mitrović et al. (2011)
16SrI-B	Gladiolus witches´ broom (GLAWC)	Netherlands	*cpn60* UT I-IB	AB599700/ b27119	Mitrović et al. (2011)
16SrI-B	Periwinkle virescence (NA)	Italy	*cpn60* UT I-IB	AB599702/ b27120	Mitrović et al. (2011)
16SrI-B	Western aster yellows (SAY)	California	*cpn60* UT I-IB	AB599707/ b27121	Mitrović et al. (2011)
16SrI-B	Mulberry dwarf phytoplasma (MD)	Japan	*cpn60* UT I-IB	AB124809/ b27122	Kakizawa et al. (2006)
16SrI-B	Onion yellows phytoplasma (OY-W)	Japan	*cpn60* UT I-IB	AB124806/ b27123	Kakizawa et al. (2006)
16SrI-B	Onion yellows phytoplasma (OY-M)	Japan	*cpn60* UT I-IB	AB124807/ b27124	Kakizawa et al. (2006)
16SrI-B	Onion yellows phytoplasma (OY-NIM)	Japan	*cpn60* UT I-IB	AB124808/ b27125	Kakizawa et al. (2006)
16SrI-B	Paulownia witches'-broom phytoplasma (PaWB)	Taiwan	*cpn60* UT I-IB	AB124810/ b27126	Kakizawa et al. (2006)

16SrI-B	Sumac witches'-broom phytoplasma (SuWB)	Japan	*cpn60* UT I-IB	AB242236/ b27127	Kakizawa et al. (2006)
16SrI-B	Porcelain vine witches'-broom (PvWB)	Korea	*cpn60* UT I-IB	AB242237/ b27128	Kakizawa et al. (2006)
16SrI-B	Onion yellows phytoplasma (OY-M)	Japan	*cpn60* UT I-IB	NC_005303/ b27129	Oshima et al. (2004)
16SrI-B	Carrot yellows phytoplasma (ca2006/9)	Serbia	*cpn60* UT I-IB	AB599709/ b27130	Mitrović et al. (2011)
16SrI-B	American aster yellows phytoplasma (AY-W)	USA	*cpn60* UT I-IB	AB599691/ b27131	Mitrović et al. (2011)
16SrI-B	Hydrangea phyllody phytoplasma (HP)	Japan	*cpn60* UT I-IB	AB738737/ b27132	Takinami et al. (2013)
16SrI-B	Aster yellows phytoplasma (AY-J) [RS]	France	*cpn60* UT I-IIB	AB599689/ b27133	Mitrović et al. (2011)
16SrI-B	Carrot yellows phytoplasma (ca2006/5)	Serbia	*cpn60* UT I-IIB	AB599711/ b27134	Mitrović et al. (2011)
16SrI-B	Maize bushy stunt phytoplasma (MBS-Ver) [RS]	Mexico	*cpn60* UT I-IIIB	KT444673/ b27135	Pérez-López et al. (2016a)
16SrI-B	Maize bushy stunt phytoplasma (MBS-Col)	Colombia	*cpn60* UT I-IIIB	AB599712/ b27136	Mitrović et al. (2011)
16SrI-B	Lethal wilt oil palm phytoplasma (OP47)	Colombia	*cpn60* UT I-IIIB	JX681023/ b27137	Alvarez et al. (2014)
16SrI-B	Mexican periwinkle proliferation (MePP-Centre)	Mexico	*cpn60* UT I-IIIB	KU145529/b27186	Unpublished
16SrI-B	Mexican periwinkle proliferation (MePP-South)	Mexico	*cpn60* UT I-IIIB	KU145530/b27187	Unpublished
16SrI-B	Maize bushy stunt phytoplasma (MBS-Pueb) [RS]	Mexico	*cpn60* UT I-IVB	KT444672/ b27138	Pérez-López et al. (2016a)
16SrI-B	Iceland poppy yellows phytoplasma (IPY) [RS]	Japan	*cpn60* UT I-VB	AB242234/ b27139	Kakizawa et al. (2006)
16SrI-B	Marguerite yellows phytoplasma (MarY)	Japan	*cpn60* UT I-VB	AB242235/ b27140	Kakizawa et al. (2006)
16SrI-B	Eggplant dwarf phytoplasma (ED) [RS]	Japan	*cpn60* UT I-VIB	AB242231/ b27141	Kakizawa et al. (2006)
16SrI-B	Tomato yellows phytoplasma (TY)	Japan	*cpn60* UT I-VIB	AB242232/ b27142	Kakizawa et al. (2006)
16SrI-B	Lettuce yellows phytoplasma (LY)	Japan	*cpn60* UT I-VIB	AB242233/ b27143	Kakizawa et al. (2006)
16SrI-C	Aster yellows phytoplasma (AY-Col) [RS]	Italy	*cpn60* UT I-IC	KJ939994/ b27144	Dumonceaux et al. (2014)
16SrI-C	Aster yellows phytoplasma (CVB)	Italy	*cpn60* UT I-IC	KJ939995/ b27145	Dumonceaux et al. (2014)
16SrI-C	Carrot yellows phytoplasma (CA)	Italy	*cpn60* UT I-IC	AB599690/ b27146	Mitrović et al. (2011)
16SrI-C	Leontodon yellows phytoplasma (LEO)	Italy	*cpn60* UT I-IC	AB599701/ b27147	Mitrović et al. (2011)
16SrI-C	Clover phyllody phytoplasma (KVF)	France	*cpn60* UT I-IC	AB599695/ b27148	Mitrović et al. (2011)
16SrI-C	Potato purple top (PPT)	France	*cpn60* UT I-IC	AB599704/b27149	Mitrović et al. (2011)
16SrI-E	Blueberry stunt phytoplasma (BbSP) [RS]	Canada	*cpn60* UT I-IE	KU523402/ b27454	Unpublished

16SrI-F	Apricot chlorotic leafroll (AY-A) [RS]	Spain	*cpn60* UT I-IF	AB599699/b27150	Mitrović et al. (2011)
16SrI-P	Populus decline phytoplasma (PopD) [RS]	Serbia	*cpn60* UT I-IP	AB599710/b27151	Mitrović et al. (2011)
**16SrII**					
16SrII-A	Peanut witches´broom (PnWB) [RS]	Taiwan	*cpn60* UT II-IA	NZ_AMWZ00000000/b27152	Wan-Chia et al. (2013)
**16SrV**					
16SrV-A	Flavescence dorée phytoplasma (FD) [RS]	France	*cpn60* UT V-IA	KJ939992/b27153	Dumonceaux et al. (2014)
16SrV-A	Palatinate grapevine yellows phytoplasma (PGY)	Germany	*cpn60* UT V-IA	KJ939991/b27154	Dumonceaux et al. (2014)
16SrV-A	Rubus stunt phytoplasma (RS)	Germany	*cpn60* UT V-IA	KJ939990/b27155	Dumonceaux et al. (2014)
**16SrVII**					
16SrVII-A	Ash yellows phytoplasma (AshY) [RS]	France	*cpn60* UT VII-IA	KJ939978/b27156	Dumonceaux et al. (2014)
**16SrIX**					
16SrIX-B	Almond witches'-broom SA213	Lebanon	*cpn60* UT IX-IB	KND62606/b27157	Quaglino et al. (2015)
16SrIX-H	*Catharanthus roseus´s* phoenicium phytoplama (Cr) [RS]	Cuba	*cpn60* UT IX-IH	KJ939989/ b27158	Dumonceaux et al. (2014); Pérez-López et al. (2014)
**16SrX**					
16SrX-A	Apple proliferation phytoplasma (AP) [RS]	Germany	*cpn60* UT X-IA	KJ939977/ b27159	Dumonceaux et al. (2014)
16SrX-A	Apple proliferation phytoplasma (AT)	Germany	*cpn60* UT X-IA	NC_011047/ b27160	Kube et al. (2008)
16SrX-C	Pear decline phytoplasma (12MG305) [RS]	Canada	*cpn60* UT X-IC	KJ940000/b27161	Dumonceaux et al. (2014)
16SrX-C	Pear decline phytoplasma (12MG316)	USA	*cpn60* UT X-IC	KJ940001/ b27162	Dumonceaux et al. (2014)
16SrX-C	Pear decline phytoplasma (PD2)	Germany	*cpn60* UT X-IC	KJ940002/ b27163	Dumonceaux et al. (2014)

16SrX-C	Pear decline phytoplasma (PD INRA)	France	*cpn60* UT X-IC	KJ940003/ b27164	Dumonceaux et al. (2014)
16SrX-C	Peach yellow leaf roll phytoplasma (PYLR)	USA	*cpn60* UT X-IC	KJ940010/ b27165	Dumonceaux et al. (2014)
16SrX-F	European stone fruit yellows phytoplasma (ESFY) [RS]	Italy	*cpn60* UT X-IF	KJ940007/ b27166	Dumonceaux et al. (2014)
16SrX-F	European stone fruit yellows phytoplasma (ESFY-INRA)	Italy	*cpn60* UT X-IF	KJ939975/ b27167	Dumonceaux et al. (2014)
**16SrXII**					
16SrXII-A	Bois noir phytoplasma (BN44948) [RS]	Germany	*cpn60* UT XII-IA	KJ939979/ b27168	Dumonceaux et al. (2014)
16SrXII-A	Bois noir phytoplasma (BN44962)	Germany	*cpn60* UT XII-IA	KJ939980/ b27169	Dumonceaux et al. (2014)
16SrXII-A	Bois noir phytoplasma (BN45660)	Germany	*cpn60* UT XII-IA	KJ939981/ b27170	Dumonceaux et al. (2014)
16SrXII-A	Bois noir phytoplasma (BN46017)	Germany	*cpn60* UT XII-IA	KJ939982/ b27171	Dumonceaux et al. (2014)
16SrXII-A	Bois noir phytoplasma (BN Lebanon)	Lebanon	*cpn60* UT XII-IA	KJ939993/ b27172	Dumonceaux et al. (2014)
16SrXII-A	Bois noir phytoplasma (BN France)	France	*cpn60* UT XII-IA	KJ940008/ b27173	Dumonceaux et al. (2014)
16SrXII-A	Bois noir phytoplasma (BN Germany)	Germany	*cpn60* UT XII-IA	KJ940006/ b27174	Dumonceaux et al. (2014)
16SrXII-A	Bois noir phytoplasma (Stol BN)	France	*cpn60* UT XII-IA	KJ940005/ b27175	Dumonceaux et al. (2014)
16SrXII-B	Cottonbush witchets´broom (CBWB)	Australia	*cpn60* UT XII-IB	NC_010544/ b27176	Tran-Nguyen et al. (2008)
16SrXII-B	Strawberry lethal yellows phytoplasma (SLY) [RS]	Australia	*cpn60* UT XII-IB	NC_021236/ b27177	Andersen et al. (2013)
**16SrXIII**					

16SrXIII-A	Mexican periwinkle virescence (MPV-S83) [RS]	Mexico	*cpn60* UT XIII-IA	KT444662/ b27178	Unpublished
16SrXIII-A	Mexican periwinkle virescence (MPV-S86)	Mexico	*cpn60* UT XIII-IA	KT444669/ b27179	Unpublished
**16SrXIV**					
16SrXIV-A	Bermuda white leaf phytoplasma (AL85/11) [RS]	Albania	*cpn60* UT XIV-IA	KF383984/ b27180	Mitrović et al. (2015)
16SrXIV-A	Bermuda white leaf phytoplasma (IT35/12)	Italy	*cpn60* UT XIV-IA	KF383982/ b27181	Mitrović et al. (2015)
16SrXIV-A	Bermuda white leaf phytoplasma (IT71/10)	Italy	*cpn60* UT XIV-IA	KF383983/ b27182	Mitrović et al. (2015)
16SrXIV-C	Bermuda white leaf phytoplasma (RS59/11) [RS]	Serbia	*cpn60* UT XIV-IC	KF383985/ b27183	Mitrović et al. (2015)
16SrXIV-C	Bermuda white leaf phytoplasma (RS123/13)	Serbia	*cpn60* UT XIV-IC	KJ000022/ b27184	Mitrović et al. (2015)
16SrXIV-C	Bermuda white leaf phytoplasma (RS306/13)	Serbia	*cpn60* UT XIV-IC	KJ000023/ b27185	Mitrović et al. (2015)
